# Development and Validation of the Workplace Affective Events Survey

**DOI:** 10.7759/cureus.46236

**Published:** 2023-09-29

**Authors:** Janhavi Devdutt, Paulomi Sudhir, Seema Mehrotra

**Affiliations:** 1 Student Counseling Services, King Abdullah University of Science and Technology (KAUST), Thuwal, SAU; 2 Clinical Psychology, National Institute of Mental Health and Neurosciences, Bengaluru, IND

**Keywords:** organizational psychology, daily events at work, affective events at work, affective events, emotions at work

## Abstract

Background

Work, a central aspect of human life, serves vital economic and social functions. There is a burgeoning interest in positive emotions in the workplace, which can enhance creativity, foster social connections, and improve problem-solving abilities. These emotions are pivotal in key organizational outcomes, including employee performance and health. Despite the extensive examination of factors like job satisfaction and workplace stressors, a knowledge gap exists regarding the everyday workplace events that influence emotions and their contribution to overall workplace emotional health. The present study introduces the Workplace Affective Events Survey (WAES), a new tool that can facilitate the advancement of research in this field.

Purpose

This study aimed to develop a tool to assess daily workplace events that lead to positive or negative emotional responses and the intensities of such responses. The study also examined the relationship between these events and the associated affect-intensities with trait affect, and social companionship at work for convergent validation.

Methodology

The tool development entailed a multi-phase approach which encompassed item generation, content validation, pre-pilot trials, and pilot testing of the WAES. Participants were entry and mid-level service sector employees aged 25-55 years. Themes generated using focus group discussions and one-to-one interviews were mapped against a known taxonomy of workplace affective events. Expert validation and pre-pilot trials helped in refining the final items. The main phase engaged 300 individuals from nine service industries across 29 organizations in an urban metropolitan city in India. WAES was administered alongside standardized measures of trait-affect and workplace social companionship.

Results

WAES subscales demonstrated acceptable reliability. Participants reported positive daily affective events more often than negative ones, with the average intensity of positive emotions surpassing that of negative emotions. Notably, trait affect scores and social companionship exhibited significant correlations with daily affective events and their intensity.

Conclusions

The WAES offers a novel tool to investigate daily emotional experiences in the workplace. The data suggest that a within-person disposition such as trait-affect might play a lesser role in generating positive affective events than contextual factors. These findings underscore the value of creating work environments that consistently nurture positive emotional experiences.

## Introduction

Work is crucial in our lives, serving economic and significant social purposes [1]. Beyond its financial implications, employment offers psychological benefits, such as time structure, opportunities for collaborative endeavors, social contact, and a sense of purpose or meaning, all of which can bolster mental well-being [2].

This research is a subset of a broader study and focuses on the development and validation of the Workplace Affective Events Survey (WAES). This tool assesses daily affective experiences in the workplace. Investigating such workplace events is vital because, according to the Affective Events Theory (AET) [3], specific incidents, referred to as ‘affective events’, trigger emotional responses. These emotions then influence attitudes and behaviors in the workplace, leading to various positive or negative outcomes [4]. Intense emotions, for instance, can negatively affect performance, compromise rational decision-making, and lead to biased judgments in uncertain situations [5]. While negative emotions and their effects at work have been extensively studied, there is an emerging focus on the role and impact of positive emotions in a work environment. Positive emotions can expand individuals’ thought and action repertoire, allowing for diverse ideas, actions, or solutions [6,7].

Consequently, positive events and the emotions they invoke can amplify creativity, resourcefulness, social bonding, problem-solving skills, and stress reduction [8,9]. Employees with positive interactions related to their jobs, superiors, and colleagues often report improved moods [10,11], enhanced self-worth, and a heightened sense of control [12,13]. Therefore, both positive and negative emotions impact crucial organizational elements, such as performance, conflict resolution, problem-solving, turnover rates, and employee health [14,15].

Given that work constitutes a significant aspect of many adults’ lives, their work experiences undoubtedly influence their overall well-being. Numerous personal and contextual factors can affect workplace emotions [16]. Overall well-being in the workplace has been associated with outcomes like job satisfaction and engagement [17-19]. Similarly, chronic workplace stressors [20,21], significant events such as workplace bullying and job strain [22,23], and job features [24-26] have been scrutinized for their impact on work-related results. However, less research has delved into specific daily occurrences that might generate emotions in the workplace and define the broader scope of affective well-being at work.

Rationale for the study and objectives

Many studies have explored the tenets of AET, predominantly using the experience sampling method to capture workplace emotions and events. While the experience sampling method can effectively capture real-time events, it poses challenges in the workplace due to its demanding nature on the participants and its potential to disrupt work activities. Our review of the literature before undertaking the study indicated that there is a dearth of structured tools/measures to systematically document these events and experiences.

Therefore, the objectives of the present study were to develop a tool to methodically record the occurrence of daily workplace events that give rise to positive/negative effects and to evaluate its reliability and convergent validity. Furthermore, the study investigates the correlation of these workplace events with variables such as age, gender, and certain work-related aspects.

Various dispositional and contextual factors intertwine to influence affective experiences [16]. This study used trait affect, which denotes stable predispositions to certain emotional states, and social companionship at work as a contextual variable, to assess convergent validity. Here, trait affect describes how respondents usually feel, and its influence on emotional reactions to workplace events has been documented [27]. On the other hand, social companionship relates to the availability of supportive relationships at work and was evaluated using the Loneliness in the Workplace scale’s Social Companionship subscale [28]. Prior research has linked workplace social support with affective experiences [29,30].

## Materials and methods

This study progressed through several phases: item generation, content validation, pre-pilot trial, and pilot trial. Throughout all phases, the sample selection criteria remained consistent. A cross-sectional, mixed methods (quantitative and qualitative) design was used for data collection.

Sample

The pilot trial sampled 300 individuals with an average age of 36.7 years (SD 8.7) and comprised 153 women and 147 men. These participants were drawn from various workplaces in Bengaluru, India. We established the following inclusion criteria for sample selection: participants should be 25-55 years old, possess a minimum of 12 years of education, be proficient in reading, writing, and speaking English, and be employed in the service sector. Additionally, the sample inclusion criteria also required that a potential participant should have at least one colleague in their work unit aside from a supervisor. Exclusion criteria consisted of those who had been at their current workplace for less than six months, engaged in part-time work (fewer than 30-35 hours weekly), operated their own businesses, or worked in the unorganized service sector with fewer than 10 staff members. We targeted participants from the service sector because it constitutes a significant portion of India’s economy, contributing to over half of India’s gross domestic product [31]. Our plan encompassed recruiting participants from nine service sectors delineated by the National Industrial Classification [32], which included information and communication, education, human health and social work activities, professional, scientific, and technical activities, retail trade, financial and insurance activities, accommodation and food service activities. The study was approved by the Institute Ethics Committee of the National Institute of Mental Health and Neurosciences, Bengaluru, India, and written informed consent was obtained from all prospective participants.

Tools

The study used three primary tools. The basic datasheet was designed to capture the participants’ basic sociodemographic and work characteristics. It covered topics such as education, duration of employment, supervisory roles, work-related stress, and more. The Positive Affect and Negative Affect Schedule (PANAS), Revised [33], is an updated version of the original PANAS scale [34,35]. It consists of 26 affect-related statements, with 13 each representing positive and negative emotions. By summing the scores for each scale separately, positive and negative affect levels can be determined. The PANAS has consistently demonstrated robust psychometric properties within Indian cohorts [33,36]. This measure can be used with different instructions for capturing emotional experiences within different time frames (e.g., last one week) or to assess the experience of emotions in general/usually/typically (trait affect). In the present study, the participants were asked to provide ratings for the items in terms of the extent to which they experienced the given emotions in general/usually. Lastly, the Social Companionship subscale of the Loneliness at Work Scale consists of seven items assessing supportive coworker relationships and dependable social alliances. Participants’ responses were measured on a seven-point Likert scale, ranging from ‘strongly agree’ (1) to ‘strongly disagree’ (7).

Procedure

We began by researching organizations in Bengaluru, India, that belonged to the nine service sectors online. After approaching 39 through various communication modes, 22 granted us permission for participant recruitment. We aimed to draw participants from at least two organizations per service sector. As this method did not fulfill our target sample size of 300, we also directly recruited participants using snowballing. These participants belonged to seven additional workplaces. Thus, the overall sample was drawn from 29 workplaces across the array of nine service sectors. Some examples of workplaces from where participants were sourced included schools, hospitals, banks, hotels, information technology companies, and retail businesses.

In our study, we adopted quota sampling. The primary variables for sample stratification were age and gender. Participants were categorized into two age brackets: 25-35 years (young adulthood) and 36-55 years (middle adulthood), and further stratified by gender within these age groups. An attempt was made to recruit an equal number of participants in each age group. Efforts were also directed at ensuring sample heterogeneity in terms of levels of work. (entry and mid-level). In the present study context, entry-level employees were those who reported to a single supervisor and did not oversee others. Conversely, mid-level employees supervised other team members and reported to a superior. The final participant selection depended on our set criteria, consent, age, and gender.

Interactions with participants were organized individually or in groups during their working hours. The study’s objectives were explained, and instructions were given before distributing the survey questionnaire. The first author conducted group administrations with seven to 11 participants when feasible. In situations where group administrations were impractical, participants could choose to complete an online or offline survey.

Data analyses

Data were coded and analyzed using the SPSS for Windows, Version 16.0 (Released 2007; SPSS Inc., Chicago, United States). Descriptive statistics like frequency, percentage, mean, and standard deviation were used. Normality of the distributions was examined using the Kolmogorov-Smirnov test (KS -Z test). Depending on normality, appropriate parametric (Independent Sample t-test, Pearson Product Moment Correlation) or non-parametric statistics (Mann-Whitney U-test, Spearman Rank Order Correlations) were used for subgroup comparisons and correlational analyses. As far as the qualitative data in the initial phase of the study is concerned, focus group discussions and interviews were audio recorded wherever consent for the same was given by the participants, or else extensive notes were made by the researcher. The audio recordings were transcribed, and content analysis was used to capture the day-to-day workplace events that generated positive and negative emotions. This process was carried out by the first author and cross-checked by the third author.

## Results

Development of the WAES

During the item generation phase, FGDs and individual interviews gathered data from a purposive sample of 31 working individuals (20 women, 11 men) who had an average age of 35 years and had been in paid employment for an average of 16.2 years. The primary objective was to understand the nature of affective experiences in the workplace, which would guide the creation of survey items. Questions explored included participants’ general perspectives on emotions at work, the variety of emotions they encountered, and specific events that prompted both positive and negative feelings.

In total, five FGDs were conducted. Of the 62 prospective participants, 22 provided written consent to participate. To ensure a comprehensive understanding of affective experiences, the study aimed to encompass a diverse group of individuals employed across multiple service industries. However, conducting FGDs with colleagues from the same workplace sometimes led to hesitation in sharing, while logistical difficulties arose when trying to bring together individuals from different workplaces. Hence one-on-one interviews were carried out with nine additional participants to supplement FGD data. The FGD and interview participants were sampled from sectors including education, research, health, finance, and design. The FGDs and interviews had similar lines of inquiry and aimed at eliciting the nature of day-to-day events at work that gave rise to positive and negative emotions.

In the subsequent phase, the taxonomy of workplace affective events created by Basch et al. [37] served as a foundational reference to classify/map the data acquired in the initial phase (Table 1).

**Table 1:** Emergence of workplace affective event categories

Workplace Affective Events Categories	Emerged in Data	Used for Item Generation
Receiving recognition	Yes	Yes
Goal achievement	Yes	Yes
Acts of colleagues	Yes	Yes
Involvement in challenging/novel tasks	Yes	Yes
Acts of management	Yes	Yes
Task problems	Yes	Yes
Lack of influence/control	Yes	Yes
Lack of goal achievement	Yes	Yes
Workload	Yes	Yes
External/physical work environment	Yes	Yes
Personal problems	Yes	Yes
Lack of recognition	Yes	Yes
Making mistakes	Yes	Yes
Goal progress	Yes	Yes
Involvement in decision-making	Yes	Yes
Involvement in planning	Yes	Yes
Involvement in problem-solving	Yes	Yes
Influence or control	No	No
Organization reputation	No	No
Disconfirmation of negative expectation	No	No
Company policies	No	No
Expression of gratitude	Yes	Yes
Competition	Yes	Yes
Working together as a team	Yes	Yes
Misunderstandings	Yes	Yes
Feedback about work	Yes	Yes
Feedback about personal attributes	Yes	Yes
Autonomy at work or lack thereof	Yes	Yes
Close associate leaving organization	Yes	No

Events related to the organization’s reputation, disconfirming negative expectations, and company policies though mentioned in the taxonomy did not fit the study’s focus on daily work events and were therefore omitted. While influence or control over colleagues was not a recurring theme in the gathered data, participants frequently mentioned autonomy at work or the lack thereof. This shifted the focus to events like the freedom to voice opinions or experiences of feeling constrained at work. Unique themes from the FGD and interview data that did not fit under predefined event categories in the taxonomy were retained for item generation to capture a broad spectrum of workplace events.

A pool of items was generated based on FGD and interview data and literature review Criteria for generating items were to include only day-to-day events at work (as opposed to major/infrequent work events) and to incorporate events that are likely to generate clear-cut positive/negative emotions. This draft was further refined by seeking feedback from three seasoned psychological and affect research experts with 10 years of experience . They assessed each item based on its clarity, relevance, and difficulty. Consequently, after considering the experts’ feedback, three items were removed, and some were reworded, leaving 115 items. These items were then reviewed by the first and the third authors independently. Items were categorized into positive and negative events . They also evaluated the items for potential overlaps and the likelihood of their occurrence ( rare/non-rare). . Differences in ratings between the two were resolved through discussions. This process removed 11 overlapping and 27 infrequent items, leaving a final tally of 77 items. for the survey. Examples of such items include ‘A colleague greeted me with a genuine smile’ and ‘A colleague offered to help me with my work.’ For every event listed in the survey, participants had to specify its frequency over the past two weeks, its emotional valence (positive or negative), and the intensity of the emotion it elicited.

Pre-Pilot Trials I and II

The draft version of the survey was shared with 33 working individuals who met the study’s inclusion and exclusion criteria. Feedback, such as concerns over the length of the questionnaire and repetitive items, led to item revisions and preliminary data analysis. When items overlapped or seemed similar in content, we removed those that the pilot sample reported less frequently. We initially categorized each event as either positive or negative. However, some participants viewed certain events as positive, while others viewed the same events as negative. We either deleted or revised these contentious items to ensure they were universally understood as positive or negative.

This review left 59 items in the final survey. We removed the rating on emotion valence to streamline the response process and lessen the burden on respondents. Instead of marking an event’s frequency, participants simply indicated its occurrence with ‘yes’ or ‘no.’ We then asked participants to rate the intensity of their emotions on a five-point Likert scale, ranging from 0 (‘Did not feel any emotion’) to 4 (‘Felt very pleasant/unpleasant’). This final survey format provided four distinct scores: positive event occurrence, positive emotion intensity, negative event occurrence, and negative emotion intensity. We conducted a second pre-pilot trial on this version with 65 individuals who fit the study’s inclusion criteria. Preliminary analyses revealed that the positive and negative emotion intensity subscales demonstrated high reliability, with α values of 0.95 and 0.89, respectively.

Main phase pilot testing

Sample Characteristics

Three hundred participants formed the main study sample. Men and women were almost equally represented in this phase, as depicted in Table 2. The sample’s average age aligned with middle adulthood, with a nearly even split between young adults (aged 25-35 years: 46%) and middle-aged adults (aged 36-55 years: 54%). Both age groups had balanced gender representations. Most participants held postgraduate degrees (65%), while 34% were graduates.

**Table 2:** Basic sociodemographic characteristics of the study sample (Main Phase; N = 300) ^a^ Extended family living together; ^b^ Consisting of two parents and their children (i.e., nuclear) PUC, pre-university college; INR, Indian rupee

Sociodemographic Data	Frequency (Percentage)
Gender	
Males	147 (49%)
Females	153 (51%)
Age group	
25-35 years	137 (46%)
36-55 years	163 (54%)
Education	
Senior secondary/PUC	4 (1%)
Graduate/Diploma	101 (34%)
Post-graduate	195 (65%)
Marital status	
Never married	74 (25%)
Married	196 (65%)
Single (Separated/Divorced/Widowed)	25 (8%)
Not reported	5 (2%)
Family type	
Joint^a^	112 (37%)
Immediate^b^	149 (50%)
Not reported	39 (13%)
Income	
<55,000 INR per month	188 (63%)
55,000-1,00,000 INR per month	63 (21%)
>1,00,000 INR per month	40 (13%)
Not reported	9 (3%)

Work Characteristics

Participants had been in paid employment for an average of 12 years and at their current job for an average of six years. The sample displayed significant variability in these figures. Slightly over half reported holding supervisory roles, as shown in Table 3. We assessed perceived stress in work, health, personal/family relationships, and finances domains using a visual analog scale (0 = ‘Not at all’ to 10 = ‘A lot’). An additional open-ended item captured other stressors. Frequently cited other concerns included family health, child-rearing challenges, commuting issues, academic pressures, career advancement, and legal problems. On average, participants rated work-related stress higher than stresses in other non-work categories, as detailed in Table 4.

**Table 3:** Work characteristics of the sample (N = 300) Abbreviations: NA, not applicable; SD, standard deviation

Characteristics	Frequency (Percentage)	Mean	SD	Range
Supervisory role (current) – Yes	167 (56%)	NA	NA	NA
Supervisory role (current) – No	129 (43%)	NA	NA	NA
Supervisory role - Not reported	4 (1%)	NA	NA	NA
Total years of paid employment	NA	11.7	8.7	0.5 – 38 years
Years in current employment	NA	5.7	7.3	0.5 – 35 years

**Table 4:** Perceived stress (N = 300) ^a^ Average stress in all non-work domains (health, relationship, financial, other) SD, standard deviation

Stressor	Minimum - Maximum		Mean (SD)	Median
	Possible	Obtained		
Work stress	0-10	0-10	4.62 (2.61)	5.00
Health stress	0-10	0-10	3.55 (2.72)	3.00
Personal/family relationship stress	0-10	0-10	3.49 (2.94)	3.00
Financial stress	0-10	0-10	3.55 (2.92)	3.00
Other stress	0-10	0-9	5.56 (2.77)	5.00
Non-work stress^a^	0-10	0-8.67	3.53 (2.29)	3.33

Descriptive Statistics of the WAES

In the main data collection phase, we divided the WAES into sections for positive and negative events. Participants were asked about events from the past two to three weeks. If an event occurred during this timeframe, they indicated ‘yes’ or ‘no.’ If the response was ‘yes,’ they further rated the emotion’s intensity on a scale from 0 (‘did not feel any emotion’) to 4 (‘felt very pleasant/unpleasant’).

The WAES’s positive and negative affect intensity scales displayed strong reliability, as shown in Table 5. Participants reported more positive events at work than negative ones. They noted 23 of the 30 positive events in the WAES, a notably higher figure than nine of the 29 negative events. The average positive emotion intensity (PEI) from these positive events (63.96 ± 27.47) also exceeded the negative emotion intensity (NEI) from negative events (19.39 ± 20.67). A significant difference was observed between these affect intensities (Z = 14.14, p < 0.001).

**Table 5:** Descriptive statistics of the WAES (N = 300) ^a^ For assessing normality of distribution; ^***^p = .000 ns, non-significant, SD, standard deviation; WAES, Workplace Affective Events Survey

Variable	Number of Items	Minimum-Maximum Possible	Minimum-Maximum Obtained	Mean (SD)	Internal Consistency (α)	K-S Z Value^a^
Positive Event Occurrence	30	0-30	2-30	22.95 (6.01)	--	2.08***
Positive Emotion Intensity		0-120	1-120	63.96 (27.45)	0.94	0.71 (ns)
Negative Event Occurrence	29	0-29	0-29	9.45 (8.36)	--	2.59***
Negative Emotion Intensity		0-116	0-97	19.39 (20.67)	0.94	3.01***

Three of the most common positive affective events at work related to interactions with colleagues, as shown in Table 6. On the other hand, three of the least common positive events also revolved around interactions with colleagues. Over 84% of participants endorsed the most common positive affective events at work. Between 29% and 56% of participants did not experience positive events tied to expressing diverse opinions in meetings, engaging in brainstorming sessions, discussing personal or work-related challenges with colleagues, sharing workloads, or receiving apologies for mistakes or misunderstandings.

**Table 6:** The most and least endorsed positive affective events at work

Positive Affective Events Reported at Work		Frequency in Sample (%)	
		Occurrence	Non-occurrence
Most Endorsed	A colleague greeted me with a genuine smile.	287 (96%)	-
	Despite difficulties I completed my task on/before time.	273 (91%)	-
	My ideas and opinions at work were heard by my colleagues.	262 (87%)	-
	A colleague asked me to join him/her for a coffee/tea break at work.	256 (85%)	-
	I was chosen for some important work responsibility.	251 (84%)	-
	My colleague thanked me for the help/support they received from me.	262 (87%)	-
Least Endorsed	I had an opportunity to express my difference of opinion freely in a meeting with colleagues/superiors.	-	88 (29%)
	A colleague wanted to listen to my difficulties (personal/work related) willingly.	-	93 (31%)
	I was involved in the process of brainstorming possible solutions to a problem that arose at work.	-	97 (32%)
	My colleague offered to help me with the extra work load thereby reducing my burden.	-	123 (41%)
	A colleague apologized to me for a mistake/misunderstanding.	-	169 (56%)

Negative affective events most frequently reported were endorsed by 40-48% of participants and pertained to management actions, colleague behaviors, and communication challenges (Table 7). Among the least reported negative events, three centered on the unfair assignment of tasks or denials. Others included difficulties resolving customer issues and perceived feelings of social exclusion at work. Nonetheless, even the least common negative event received endorsement from 20% of participants.

**Table 7:** The most and least endorsed negative affective events at work

Negative Affective Events Reported at Work		Frequency in Sample (%)	
		Occurrence	Non-occurrence
Most Endorsed	I remember an incident (in the past 2-3 weeks) when I was treated unfairly by someone at work.	143 (48%)	-
	A co-worker did not do what was expected of him/her and I had to do that.	142 (47%)	-
	I heard a colleague gossiping/talking negatively about me at the workplace.	128 (43%)	-
	I made a mistake at work.	126 (42%)	-
	My colleague/superior/junior did not try to understand what I was trying to convey.	119 (40%)	-
Least Endorsed	I was assigned a task that was well below my qualification/expertise.	-	224 (75%)
	I was denied leave at work when I thought that it could have been granted.	-	228 (76%)
	I felt that the task given was beyond my skill/capacity.	-	231 (77%)
	I was not able to solve a client’s/customer’s problem/issue at work despite trying.	-	231 (77%)
	I was excluded from a social meeting/group/conversation at work.	-	239 (80%)

Convergent Validity

When examining the correlations between WAES scores and trait affect and social companionship, respectively, we found that trait positive affect correlated more strongly with the intensity of positive emotions at work than with their occurrence. Additionally, it had a weak but significant negative correlation with both the occurrence and intensity of negative affective events. Trait negative affect correlated equally strongly with both negative event occurrences and negative emotion intensities. Positive emotion intensity had a stronger positive relationship with social companionship at work than positive event occurrences. Conversely, both negative emotion intensity and negative event occurrence correlated negatively with social companionship (Table 8).

**Table 8:** Correlations of WAES scores with trait affect and social companionship ^**^p < .01; Spearman correlation coefficients; ^⸸^Pearson correlation coefficients WAES, Workplace Affective Events Survey

Variables	Positive Event Occurrence	Positive Emotion Intensity	Negative Event Occurrence	Negative Emotion Intensity
Trait Positive Affect	.15^**^	.30^**⸸^	-.19^**^	-.16^**^
Trait Negative Affect	-.05	-.10	.41^**^	.42^**^
Social Companionship	.17^**^	.37^**⸸^	-.23^**^	-.19^**^

Association of WAES Scores with Age, Gender, and Work-Related ariables

There were no gender differences on the WAES scores, as illustrated in Table 9. Compared by age, young adults showed significantly higher scores for negative event occurrence and negative emotion intensity than their middle-aged counterparts (Table 10). However, no difference was observed between these age groups in terms of positive affective event occurrences or intensities.

**Table 9:** Gender differences in WAES scores ^a^ Independent t-test value WAES, Workplace Affective Events Survey; PEO, positive event occurrence; PEI, positive emotion intensity; NEO, negative event occurrence; NEI, negative emotion intensity; SD, standard deviation; NA, not applicable

Variables	Men (n = 147)		Women (n = 153)		Mann-Whitney U (Z)/t-value	P-value
	Mean (SD)	Median	Mean (SD)	Median		
PEO	22.63 (6.14)	24.00	23.25 (5.89)	24.00	0.92	.36
PEI	62.17 (28.52)	NA	65.69 (26.41)	NA	1.11^a^	.27
NEO	9.78 (8.66)	7.00	9.17 (8.07)	7.00	0.49	.62
NEI	19.72 (20.43)	12.00	19.07 (20.94)	11.00	0.39	.69

**Table 10:** Comparison of WAES scores across age categories ^a^ Independent t-test value WAES, Workplace Affective Events Survey; PEO, positive event occurrence; PEI, positive emotion intensity; NEO, negative event occurrence; NEI, negative emotion intensity; SD, standard deviation; NA, not applicable.

Variables	Young adults (n = 137)		Middle-age adults (n = 163)		Mann-Whitney U (Z) value/t-value	P-value
	Mean (SD)	Median	Mean (SD)	Median		
PEO	23.67 (5.69)	24.00	22.32 (6.25)	23.00	1.88	.06
PEI	66.99 (26.74)	NA	61.41 (27.90)	NA	1.76^a^	.08
NEO	10.79 (8.42)	9.00	8.36 (8.17)	6.00	2.94	.003
NEI	21.35 (21.48)	14.00	17.75 (19.88)	10.00	2.09	.036

WAES scores showed low but significant associations with work and non-work stress (Table 11). Lower levels of work stress were significantly associated with reports of a greater number of positive event occurrences and higher positive emotion intensity at work. Non-work stress was not related to positive event variables at work. However, higher work, as well as non-work stress, were correlated with higher negative event occurrences and negative emotion intensity at work.

**Table 11:** Correlations of duration of current employment and work and non-work stress with WAES scores ^*^p < .05; Spearman correlation coefficients; ^***^p < .001; Spearman correlation coefficients; ^⸸^Pearson correlation coefficients WAES, Workplace Affective Events Survey; PEO, positive event occurrence; PEI, positive emotion intensity; NEO, negative event occurrence; NEI, negative emotion intensity

Variables	Duration of Current Employment	Work Stress	Non-Work Stress
PEO	-.02	-.12^*^	-.04
PEI	-.05	-.16^*^	-.04^⸸^
NEO	-.06	.29^***^	.24^***^
NEI	-.05	.33^***^	.26^***^

## Discussion

The current study aimed to develop a tool to examine daily affective events at work. While the nature of work can vary significantly across different service activities, our goal was to identify affective events at work with potential relevance across various industries in the service sector. Our sample displayed enough diversity to produce findings that might largely generalize to English-speaking urban Indian adults in their young to middle adulthood years who are employed in the service sector.

Nature of positive and negative affective events at work

Our results align with prior research, such as the Gallup Poll survey [38] that investigated daily affective events and found a higher frequency of positive events than negative ones in workplaces [10,39-41].

Our study also found a notable difference between average state positive and negative affect intensities (p<0.001), echoing Elfering et al.’s findings [41]. Specifically, Elfering et al. also reported mean state positive affect to be higher than the mean state negative affect assessed in the context of workplace experiences [41]. Our research centered on daily micro-events rather than major or chronic work stressors. People might view such micro-events as normal or downplay their importance, especially when larger issues are in focus. Consequently, this could reduce the average intensity of negative emotions stemming from these daily workplace events. Such pre-emptive responses, influenced by appraisal processes, can lessen the effects of negative workplace events by reducing the intensity of emotional reactions [42-44]. According to the AET, employing emotion-focused coping strategies, such as denial or cognitive and behavioral disengagement from the event, helps individuals direct their cognitive and behavioral resources toward completing tasks, proving particularly beneficial in a work environment [3]. Additionally, the mobilization-minimization hypothesis suggests that negative events initially elicit a robust physiological, cognitive, and behavioral response, followed quickly by mechanisms that mitigate their impact [45]. Examples include decreased recall of negative compared to positive events, resisting negative moods, reinterpreting negative occurrences to maintain positive self-views, and societal minimization or denial of negative events [45]. These mechanisms can curtail associated negative affective states.

Three of the most commonly endorsed positive affective events were associated with interactions or actions of colleagues, a finding that resonates with prior studies [37,39]. The most commonly occurring negative events were reported by almost half the participants. These encompassed issues like unfair treatment, errors, communication challenges, and negative colleague behaviors, such as gossip or the participant having to complete a colleague’s tasks. Past research has indicated that significant workplace events often pertain to fairness assessments, work duties, and interactions with supervisors or peers [46]. Basch et al. identified managerial actions, task challenges, and errors as frequent triggers for negative emotions at work [37]. The prevalence of interactional aspects in both positive and negative events underscores the importance of relational factors in shaping emotional experiences at work. This prominence may be more pronounced in Eastern or collective cultures like India, where upholding social order and communal harmony is a priority [47]. Notably, even the least commonly reported negative affective event was acknowledged by 20% of participants, and the least common positive event by 44%. This indicates that all WAES items resonated with at least 20% of participants, bolstering the tool’s relevance and applicability.

Trait affect and affective experiences at work

High trait positive affect refers to a dispositional tendency toward experiencing positive emotions more frequently [48]. This predisposition explains the observed associations in our study: individuals with high scores on trait positive affect not only had more frequent positive events but also experienced greater positive affect intensity. Furthermore, this inclination might also explain the reduced reports of negative event occurrences. People with positive affective traits tend to use more adaptive coping and emotion regulation strategies [49-51]. Such strategies could explain their reduced negative affect intensity (NEI) and a more positive perception of work events [27]. In contrast, Nelis et al. found that those with high trait negative affect often employ dampening strategies [52]. Negative emotions can limit our focus and constrain our cognitive and behavioral reactions. This might elucidate the observed positive relationship between trait negative affect, negative event occurrences, and negative emotion intensity at work. Moreover, individuals with high trait negative affect might experience prolonged mood recovery or repair processes, potentially affecting their overall psychological well-being [53].

On the other hand, the relative independence (low correlation) of trait positive affect and daily positive affective experiences at work highlights that the generation of positive affective events and ensuing positive affect may be less driven by dispositional factors such as trait affect and impacted to a greater extent by contextual variables at work. This, in turn, draws attention toward the scope for organizational-level interventions and practices that can enhance well-being in the workplace through the promotion of work environments that nurture day-to-day positive affective experiences.

Social companionship and affective experiences in the workplace

Our study found that participants with greater social companionship at work reported a higher frequency of both positive event occurrences and PEI. Conversely, they reported fewer negative events and a weaker negative emotional response. Authayarat et al. highlighted the importance of cooperative team spirit and cohesive coworker relationships as precursors to positive workplace experiences [54]. Having supportive colleagues enhances an individual’s personal resources to combat stress. Social support can modulate emotional responses through empathetic listening, perspective-sharing, offering alternative solutions, or simply serving as a positive diversion [55,56]. These dynamics might explain the observed negative correlation between social companionship, negative event occurrences, and negative emotional intensity at work. The observed correlations between trait affect, social companionship, and our new measure support its convergent validity.

Affective experiences at work, sociodemographic, and work characteristics

In our sample, young adults reported significantly greater negative events and associated negative emotion intensity than their middle-aged counterparts. This observation might arise from the young adults displaying a significantly higher trait negative affect, a pattern observed in previous research [57,58]. Both work-related, and non-work stress ratings correlated positively with negative affective events and their associated intensity. It is conceivable that these variables have a bidirectional relationship: negative events can exacerbate work stress, which could influence the occurrence or experience of such events. Conversely, the presence of positive affective events might have some buffering impact on work stress. Future research should explore these hypotheses. While the duration of current employment showed no relation to affective event occurrences and intensities, employees with varying tenures might have qualitatively distinct emotional experiences at work.

Strengths and limitations of the study

The present study is one of the few studies, particularly from India, that examine day-to-day affective events at the workplace. The development of a theory-based tool to systematically measure such events, using stakeholder observations and prior research for item generation, and utilizing a diverse sample from 29 organizations across nine service sectors for pilot evaluation of the tool are some of the strengths of the study.

Our study had some notable limitations. It focused solely on day-to-day affective events, excluding chronic stressors or significant but infrequent work-related events. We used snowballing in addition to organizational recruitment, potentially introducing sampling bias. The results are primarily applicable to English-speaking, entry and mid-level service sector employees. Given the study’s cross-sectional design, we cannot infer causality between variables. Furthermore, the psychometric properties of our developed tool require confirmation through studies using diverse samples.

## Conclusions

Our study describes the development and psychometric properties of a new tool to examine daily emotional experiences at work. The WAES holds promise as a valuable means of assessment for capturing day-to day workplace affective events that are likely to have a bearing on emotional well-being and other outcomes. Moreover, the pattern of relationships observed between workplace affective events, trait affect, and social companionship have offered several leads for further research in the field.
